# Behaviour Change Techniques in Weight Gain Prevention Interventions in Adults of Reproductive Age: Meta-Analysis and Meta-Regression

**DOI:** 10.3390/nu14010209

**Published:** 2022-01-03

**Authors:** Mamaru Ayenew Awoke, Cheryce L. Harrison, Julie Martin, Marie L. Misso, Siew Lim, Lisa J. Moran

**Affiliations:** Monash Centre for Health Research and Implementation (MCHRI), School of Public Health and Preventive Medicine, Monash University, Clayton, VIC 3168, Australia; mamaru.awoke@monash.edu (M.A.A.); cheryce.harrison@monash.edu (C.L.H.); juliechristine34@yahoo.com (J.M.); Marie.Misso@monash.edu (M.L.M.); siew.lim1@monash.edu (S.L.)

**Keywords:** behaviour change techniques, weight gain prevention, reproductive age, meta-analysis, meta-regression

## Abstract

Weight gain prevention interventions are likely to be more effective with the inclusion of behaviour change techniques. However, evidence on which behaviour change techniques (BCT) are most effective for preventing weight gain and improving lifestyle (diet and physical activity) is limited, especially in reproductive-aged adults. This meta-analysis and meta-regression aimed to identify BCT associated with changes in weight, energy intake and physical activity in reproductive-aged adults. BCT were identified using the BCT Taxonomy (v1) from each intervention. Meta-regression analyses were used to identify BCT associated with change in weight, energy intake and physical activity. Thirty-four articles were included with twenty-nine articles for the meta-analysis. Forty-three of the ninety-three possible BCT listed in the taxonomy were identified in the included studies. *Feedback on behaviour* and *Graded tasks* were significantly associated with less weight gain, and *Review behaviour goals* was significantly associated with lower energy intake. No individual BCT were significantly associated with physical activity. Our analysis provides further evidence for which BCT are most effective in weight gain prevention interventions. The findings support that the use of key BCT within interventions can contribute to successful weight gain prevention in adults of reproductive age.

## 1. Introduction

Obesity is a pressing global health challenge. The prevalence of overweight and obesity affect one-third of the world’s population and are escalating globally [1]. Both men and women of reproductive age are at increasing risk of longitudinal weight gain and development of obesity [2,3] with longitudinal data reporting they gained 0.5–0.8 kg per year [4,5]. Furthermore, women of reproductive age are at a particularly higher risk of weight gain and obesity exacerbated by excess gestational weight gain and postpartum weight retention. For example, reproductive age women in Australia had an average weight gain of 6.3 kg over 10 years [6] with this rate of weight gain greater in women 18–50 years (0.4–0.7 kg/year) compared to women above 50 years (0.2–0.5 kg/year) [7]. In addition to increasing the risk of obesity, weight gain in adults is associated with increased risk of various chronic diseases including type 2 diabetes, hypertension, cardiovascular diseases and cancer [8,9] and an overall increased risk of mortality [10].

Prevention of weight gain is considered less expensive, more feasible and effective than obesity treatment [11]. Once established, obesity treatment is more intensive, costly and largely unsustainable [12,13]. In response to this challenge, there is a need to consider a greater emphasis on weight gain prevention to curb the rising prevalence of overweight and obesity [14,15]. A recent meta-analysis of 29 studies by our group assessed the efficacy of lifestyle interventions for the prevention of weight gain in 37, 407 adults [16]. Overall, lifestyle interventions were effective in preventing weight gain in adults aged 18–50 years (MD −1.15 kg; 95% CI −1.50, −0.80) compared to control [16]. Interventions were effective for both women and men. The impact of the interventions was also more pronounced in non-obese adults and for prescriptive compared to non-prescriptive interventions. However, behaviour change strategies associated with the intervention effectiveness remain to be identified.

Lifestyle interventions are often complex and involve multiple components also known as active ingredients designed to change behaviour [17]. A behaviour change technique (BCT) has been previously defined as an “observable, replicable, and irreducible component of an intervention designed to alter or redirect causal processes that regulate behaviour; that is, a BCT is proposed to be an ‘active ingredient’” [18]. A taxonomy of behaviour change technique (BCTTv1) has been developed for better understanding of complex interventions and identification of active ingredients of interventions that contribute to positive behaviour change. This taxonomy by Michie et al. [18] provides a standardized list of 93 BCT labels and detailed definitions. For example, some key BCTs are: *Goal setting behaviour* (e.g., eat 2 serves of fruit and 5 serves of vegetables each day, aim 8000–10,000 steps per day), *Problem solving* (e.g., identify barriers or facilitator for change, relapse prevention), *Self-monitoring of behaviour* (e.g., regular self-weighing, using pedometer or diary), *Review behavioural goals* (email or written feedback on energy intake and physical activity), *Social support* (unspecified, (encouraged to walk with friends or join compatible local group programs), *Graded tasks* (encourage a gradual increase in physical activity levels-working towards 150–300 min per week) and *Behavioural practice/rehearsal* (e.g., exercise classes with role play). Several previous meta-regression analyses have investigated BCTs associated with change in diet, physical activity and weight [19,20,21,22,23]. Several reviews have also identified effective BCTS within lifestyle interventions to improve outcomes in diet [24] and weight [25,26] using percentage effectiveness ratios and have reported that interventions are likely to be more effective with the inclusion of BCTs such as self-monitoring, goal setting and social support. These studies, however, have focused on specific population groups including younger adults [24,25], pregnant women [23], postpartum women [20] and participants with chronic conditions [19] which limits the generalizability of these findings to the broader population of adults of reproductive age who experience greater longitudinal weight gain. To date, no previous studies have evaluated BCTs associated with interventions specifically targeting weight gain prevention in adults of reproductive age (18–50 years); therefore, a greater understanding of specific BCTs or combination of BCTs associated with weight gain prevention and improvements in lifestyle outcomes is required to guide future intervention development. This study aims to identify the BCTs associated with change in weight, energy intake and physical activity in adults of reproductive age.

## 2. Methods

### 2.1. Protocol and Registration

This meta-analysis was reported according to the Preferred Reporting Items for Systematic Reviews and Meta-Analyses statement [27]. The review protocol was registered with PROSPERO (registration number CRD42018114156). This work is part of our recent published systematic review and meta-analysis of lifestyle intervention of randomized controlled trials (RCTs) for preventing weight gain in adults aged 18–50 years [16]. Here, we present a secondary analysis to identify the BCTs associated with change in weight, energy intake and physical activity. 

### 2.2. Data Sources and Searches

Complete search strategies used in electronic databases, study selection, eligibility criteria, data extraction process and risk of bias assessments are reported in detail in the previous systematic review [16]. A systematic literature search was conducted with no time limit, inclusive to May 2020. Briefly, we included RCTs published in English that recruited men and women aged between 18 to 50 years, that exclusively aimed to prevent weight gain with lifestyle intervention (incorporating diet, physical activity and/or behaviour change strategies) of any duration compared with no/minimal intervention (waiting list, materials or information only interventions) and reported a weight or BMI (weight (kg)/height (m^2^)) following intervention as either a change score or endpoint value. Adults aged 18–50 were defined as reproductive age as although females under 18 and males over 50 can reproduce, it is recognized that fertility is suboptimal in older males [28] and that there are biological and social ramifications of pregnancies in women under 18 [29]. We used study level data for the outcome of weight, diet and physical activity from our previous systematic review and meta-analysis of randomized controlled lifestyle interventions to prevent weight gain [16]. Overall, 29 studies across 34 publications were included. Results including detailed description of included studies, intervention effectiveness for weight, physical activity and energy intake outcomes as well as risk of bias are reported in detail in our previous systematic review [16]. In brief, lifestyle interventions resulted in significant reductions in weight (MD −1.15 kg, 95% CI −1.48, −0.81, 29 studies, 11874 participants, *I*^2^ = 35.83%, *p* < 0.001), energy intake (MD −111.21 kcal/day, 95% CI −115.44, −106.97, 13 studies, 4207 participants, *I*^2^ = 87%, *p* < 0.001) and significant increases in physical activity levels (MD 71.75 MET-min/week, 95%CI 22.72, 120.77, 6 studies, 1329 participants, *I*^2^ = 0%, *p* = 0.004) [16]. The majority (*n* = 15) of studies were classified as moderate risk of bias [16].

### 2.3. BCTs Coding

We used the BCTTv1 [18] to identify BCTs utilized within the lifestyle interventions. Intervention descriptions of each study were reviewed and coded as presence or absence of the 93 BCTs in the taxonomy. We also referred to intervention protocols and Supplementary Materials associated with the studies and coded these for BCTs. As stated in previous systematic reviews and meta-regressions of behaviour strategies [20,22], both the intervention and control groups were coded and only BCTs that were present in the intervention group and absent in the control group were included in the analyses. BCTs were coded independently by three reviewers who have completed the BCTTv1 online training course (http://www.bct-taxonomy.com/, accessed on 23 January, 2020). Each study was independently coded by two reviewers, in which one reviewer (M.A.A) independently coded all intervention descriptions in studies and the other two reviewers (L.M. and S.L. who are dietitians with experience in lifestyle intervention development) independently coded 50% of all studies. Discrepancies were resolved by consensus in discussion with all reviewers.

### 2.4. Data Synthesis and Analysis

Data analysis methods are previously reported in the original systematic review [16]. Briefly, outcomes were pooled using the inverse variance weighted random-effects meta-analysis with the restricted maximum-likelihood estimator and expressed as mean differences (MDs) for weight (kg) and energy intake (kilocalories) with 95% confidence intervals. While we report physical outcome only for six studies reported on similar scales (MET-min/week) in our previous paper [16], here, we analyzed studies reported on different scales which can be combined as standardized mean differences (SMDs) (calculated using Hedges’ (g)) with 95% confidence interval. This was to maximize the sample size to provide sufficient power to perform meta-regression of BCTs where at least 10 studies are required. Chi-square tests were used to examine heterogeneity between studies with *p* < 0.1 considered statistically significant. The degree of inconsistency between studies was assessed using *I*^2^ with values ≥ 25%, ≥ 50%, and ≥ 75% indicating moderate, substantial and high heterogeneity, respectively [30].Publication bias was assessed with the funnel plot and Egger’s test for meta-analyses. 

### 2.5. Analysis of BCTs: Meta-Regression and Percentage Effectiveness Ratio

The total number of BCTs used per study were calculated as the sum of BCTs that were present in the intervention but not in the control group. For meta-regression and percentage effectiveness ratio, BCTs were included in the analysis if they were present in three or more studies to minimize the impact of single studies or avoid inflation of results (i.e., to reduce type−1 error) [19,31]. Here, we used two approaches to analyse BCTs and results from both methods of analyses were triangulated to increase robustness of the findings. Percentage effectiveness ratio is descriptive in nature and has the advantage of being able to identify most BCTs that have the potential to be effective. However, it may have low specificity due to its binary nature of categorization (effective/non effective), potentially including large numbers of BCTs that may only have small contributions to effectiveness but are frequently included in intervention components [32]. Meta-regressions, on the other hand, are able to detect effects that are too small to be picked up in individual studies, but they require a large number of studies and a substantial heterogeneity between studies to detect associations. 

Random effect meta-regression analyses with restricted maximum likelihood estimation were conducted to explore the associations between BCTs and changes in weight, energy intake and physical activity. Adjusted *R*^2^ was used as a measure of variance accounted for by the covariates. A series of univariable meta-regression analyses were performed to explore the effect of individual BCTs, the total number of BCTs and number of BCTs congruent with control theory (i.e., all BCTs under *Goals and planning* and *Feedback and monitoring* group) [20] on intervention effect. The group of BCTs congruent with control theory were considered here as it has been found to be associated with greater effect sizes in weight loss with lifestyle interventions in previous meta-regressions [19,20].

Additionally, a descriptive analysis of BCTs was conducted using ‘percentage effectiveness ratio’ as described in previous reviews [24,25]. Firstly, studies were categorized as effective (a significant difference in outcomes between intervention and control groups) or non-effective (no significant differences in outcomes between groups). BCTs utilized in effective and non-effective interventions were identified. The percentage effectiveness ratio was calculated as the ratio of the number of times each BCT was identified in an effective study divided by the number of times it was a component of all studies, including in non-effective trials. BCTs with percentage effectiveness ratio >50% were considered a component of effective interventions [25]. All statistical analyses were performed with STATA statistical software version 16.1 (StataCorp, College Station, TX, USA).

## 3. Results

### 3.1. Study Selection and Intervention Efficacy Overview

Study selection and screening process are shown in Figure S1 and the intervention and comparator characteristics of included studies are shown in Table S1. As reported previously, 29 studies across 34 publications were included for weight, 13 studies for energy intake and 17 studies for physical activity. Most studies involved both male and female (*n* = 17) participants, were conducted in a community settings (*n* = 18) and utilized a mixed diet and physical activity intervention (*n* = 14 studies) or behaviour change approach (*n* = 17 studies) [16]. Intervention delivery was predominantly face-to-face group sessions (*n* = 12) with median intervention duration of 9 months. Here, combining studies that reported physical activity on different scales, the intervention effect remained significant on physical activity levels (SMD 0.13, 95% CI −0.05, 0.31, 17 studies, 4496 participants, *I*^2^ = 80.77%, *p* < 0.001) (Figure S2).

### 3.2. BCT Analysis

**Figure 1:**
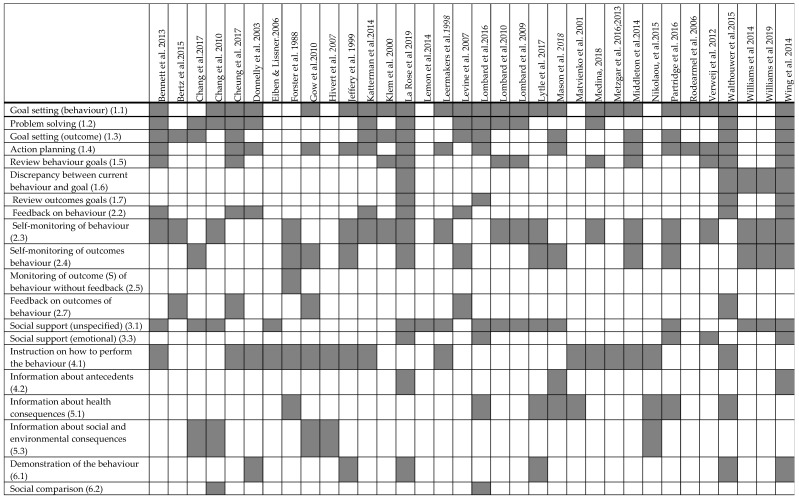

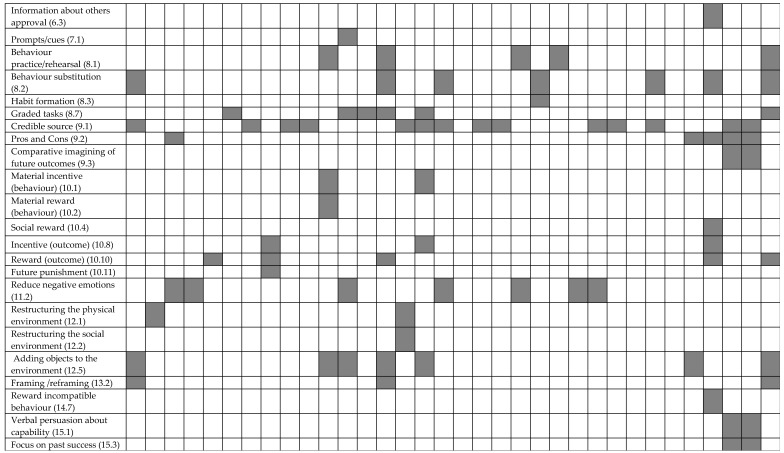
Identified behaviour change strategies in lifestyle interventions from the included studies (*n* = 34).

BCTs identified within intervention descriptions of each study have been published before [16]. Of 93 possible BCTs in the taxonomy, a total 43 BCTs unique to the intervention group were coded in the interventions (Figure 1). The number of BCTs per study ranged from 2 to 20, with an average of eight BCTs per study. The five most frequently coded BCTs were *Goal setting behaviour* (in 24 studies), *Self-monitoring of behaviour* (in 19 studies), *Action planning* (in 16 studies), *Social support (unspecified)* (16 studies) and *Instruction on how to perform the behaviour* (16 studies). 

The associations between BCTs and changes in weight, energy intake and physical activity are shown in Table 1. *Feedback on behaviour* and *Graded tasks* were significantly associated with reduced weight gain (Table 1). *Review behaviour goals* was significantly associated with a greater decrease in energy intake (Table 1). No individual BCT was significantly associated with physical activity outcomes (Table 1). Both the total number of behaviour strategies and BCTs congruent with control theory were not significantly associated with weight, energy intake or physical activity (Table 1).

A summary of BCTs identified in effective and non-effective interventions for changes in weight, energy intake and physical activity are shown in Table 2. There were 23 BCTs identified in at least three studies for weight with 18 BCTs having a percentage effectiveness ratio >50% (Table 2). For energy intake, 16 BCTs were identified in at least three studies with 9 BCTS having a percentage effectiveness ratio >50% (Table 2). For physical activity, 19 BCTs were identified in at least three studies and no BCTs showed an effectiveness ratio >50% (Table 2).

## 4. Discussion

This meta-analysis and meta-regression assessed for the first time BCTs within lifestyle intervention targeting weight gain prevention in healthy reproductive-age adult populations. As previously reported, weight gain prevention interventions prevented weight gain (1.15 kg), reduced energy intake (−111.21 kcal/day) and improved physical activity (71.75 MET-min/week) compared with controls [16]. We extended this work to report the effective BCTs associated with change in weight, diet and physical activity. While analysis from percentage effectiveness ratios suggest a number of BCTs are effective intervention components for reducing weight and energy intake, only *Feedback on behaviour* and *Graded tasks* were associated with weight and *Review behaviour goal(s)* associated with energy intake in meta-regression. No individual BCT was significantly associated with physical activity outcomes as a percentage effectiveness ratio or in meta-regression. The total number of BCTs and strategies congruent with Control Theory were not associated with any of outcomes.

A number of BCTs had a percentage effectiveness ratio >50% for weight and energy intake, but not for physical activity, with most of these related to self-regulation strategies (e.g., *Goal setting outcome*, *Review behaviour goals*, *Self-monitoring outcome(s) of behaviour,* *Feedback on behaviour*). This is consistent with a previous systematic review of electronic health interventions in young adults reporting self-regulation skills such as *Goal setting, Self-monitoring* and *Social support* were key strategies for weight gain prevention [33]. Self-regulation related BCTs were also previously associated with effective interventions for reducing energy intake in adults with obesity and chronic conditions [19,21,22]. Furthermore, interventions including *Self-monitoring* were associated with greater weight reduction in postpartum women [34], in children [35] and in adults with obesity and chronic conditions [19], although this is not consistently reported [36]. These inconsistent findings may be related to variations in methodology including BCT taxonomy used (e.g., 26-item CALO-RE taxonomy [37], redefined 40-item CALO-RE taxonomy [38], BCTT v1), population studied (young adults, postpartum women, adults with obesity and chronic conditions) and method of BCT analysis (e.g., meta-regression, percentage effectiveness ratio, Meta-CART analysis).

On meta-regression, *Feedback on behaviour* and *Graded tasks* were significantly associated with reduced weight gain. Studies evaluating specific BCTs or combination of BCTs within lifestyle interventions aimed at preventing weight gain, instead of weight management in general, in adults of reproductive age are limited. A recent review by Ashton et al. [25] identified *Goal-setting (outcome)* as an effective component for weight gain prevention interventions in young adults using percentage effectiveness ratios. We extend these findings by broadening the population studied to adults aged 18–50 years and by including a meta-regression analysis, which investigates the association between BCTs and effect sizes of intervention outcomes [21]. While the past finding on *Goal-setting (outcome)* was confirmed from our percentage effectiveness ratio analyses [25], this was not supported by the meta-regression in the current study.

In contrast to these results and previous findings on BCTs for weight gain prevention, meta regression analysis targeting weight loss interventions in adults with obesity (aged 40 years and above) reported that BCTs including *Provision of instructions*, *Self-monitoring of behaviour* and *Relapse prevention* were associated with greater weight loss [19]. None of these BCTs were associated with effect sizes of change in weight in current study. Most common BCTs associated with weight loss or weight management in previous reviews were within the BCT group of *Goals and planning* (e.g., *Goal setting, Problem solving, Action planning*) or *Feedback and monitoring* (e.g., *Self-monitoring, Personalized feedback*) [19,39]. In the current review only *Feedback on behaviour* within the *Feedback and Monitoring* BCT group was associated with weight change. These differences may be related to the fact that interventions targeting weight loss tend to be more prescriptive and intensive [40] than weight gain prevention interventions and, therefore, involve unique or distinct BCTs. Further research is needed to confirm this.

We report that *Review behaviour goal(s)* was significantly associated with a greater reduction in energy intake which is consistent with previous meta-regression analysis targeting healthy eating behaviour in adults [21]. A prior meta-regression in weight loss interventions in postpartum women reported several BCTs under *Goals and planning* BCT group (e.g., *Goal-setting of outcome*, *Problem-solving*, *Reviewing outcome goal*) and *Feedback and monitoring* BCT group (e.g., *Feedback on behaviour*, *Self-monitoring of behaviour*) were associated with greater decreases in energy intake [20]. However, only *Review behaviour goals* from the *Goals and planning* BCT group was associated with energy intake in the current study targeting weight gain prevention interventions. This indicates that *Review behaviour goal(s)* can be one of the active ingredients in interventions aiming at reducing energy intake with potential benefits for weight gain prevention. Fewer BCTs identified for weight gain prevention may again reflect the fact that prevention of weight gain requires a smaller change in energy intake [41] than weight loss (cumulative energy deficit of 3500 kcal per 0.5 kg weight loss) [42] and lifestyle interventions to prevent weight gain may, therefore, include less or distinct BCTs.

We did not find any individual BCTs significantly associated with physical activity. This is consistent with prior research in postpartum women [20] and in adults with obesity and obesity-related comorbidities [19] using meta-regression. In contrast, another review reported several BCTs including action planning, providing instruction and reinforcing effort towards behaviour were associated with physical activity in older adults [43]. However, this study used a different method of BCT identification instead of meta-regression and older version of BCT taxonomy that limits the comparison of findings. *Providing feedback*, *review of feedback* and *relapse prevention* have been previously suggested in a meta-review as effective BCTs in changing physical activity levels albeit with inconsistent findings [36]. However, specific BCTs or components of intervention to guide changes in physical activity and subsequently weight remain unclear in weight gain prevention trials. As weight gain prevention can be achieved by optimizing both diet and physical activity [44] and there are independent health benefits in engaging in a healthy diet and regular physical activity [45,46], there is a need for further research on identifying BCTs associated with physical activity in healthy adult populations of reproductive age.

Here, we also report that the total number of BCTs used in lifestyle interventions was not associated with weight, energy intake or physical activity consistent with prior research by Dombrowski et al. among adults in weight loss intervention using older version of the BCT taxonomy [19]. Similarly, another meta-regression found no significant association between number of BCTs and vegetable and fruit intake in adults of retirement age [47]. In contrast, a meta-regression in weight loss interventions in postpartum women found significant association between increased number of BCTs and decreases in energy but not weight and physical activity [20,22]. The exact reason for these inconsistent findings is not clear, although this may be related to the population studied. Using more BCTs may lead better outcomes for energy intake or eating behaviour in postpartum women [48] as they have additional barriers for healthy eating relating to their specific life stage. However, using a greater number of BCTS in interventions may increase the complexity of interventions [49], which may contribute to challenges in broader implementation [49]. Future research should determine the benefits of using effective types of numbers of BCTs or parsimonious set of BCTs for both efficacy and successful implementation [21].

The strength of this review were: (1) use of the most recent validated BCT taxonomy (BCTTv1) for BCTs coding; (2) coding of BCTs by trained three independent reviewers who also had experience in developing lifestyle interventions; use of rigorous method of BCT analysis using both percentage effectiveness ratio as explorative analysis and meta-regression to identify BCTs associated with intervention effectiveness effect size following previous recommendations [19,21]; focusing on weight gain prevention interventions in adults of reproductive age distinguishing it from previous reviews [22,24,25] through BCT taxonomy used, target outcomes and population studied.

However, the current study has several limitations. Firstly, our search was restricted to studies published in English language only. Secondly, most of the included studies provided insufficient studies in assessing the risk of bias. Thirdly, BCTs coding (presence or absence) depend on the description of interventions details reported in RCTs with an insufficiently detailed methodology precluding accurate analysis. This limitation was minimized through reviewing and coding methodology protocols and Supplementary Files or supporting documents for included studies. Fourth, we were unable to assess the relative effectiveness of different BCTs between men and women due to insufficient numbers of studies that present data sex differences. Fifth, the analysis was limited to the effect of individual BCTs and, therefore, does not show the effect of combination of BCTs. While this can be done using a meta-classification and regression trees (Meta-CART) analysis [50,51], we were unable to perform this due to insufficient number of studies. Lastly, this study was limited by the small number of studies which may reduce the chance of detecting the true effect with meta-regression. The lack of a significant effect of BCTs observed here may not indicate that these specific techniques are not important components of lifestyle interventions for weight gain prevention.

## 5. Conclusions

This meta-regression analysis showed that *Feedback on behaviour* and *Graded tasks* were associated with effect sizes in weight and *Review behaviour goal(s)* was associated with reduced energy intake. Further studies are required to confirm key BCTs associated with physical activity and to evaluate the interactive and synergetic effect of BCTs for intervention effectiveness.

## Figures and Tables

**Table 1:** Univariable meta-regression results for weight and energy intake by behaviour change techniques.

Behaviour Change Strategies	Weight (*n* = 29)		Energy Intake (*n* = 13)				Physical Activity (*n* = 17)		
	β (95%CI)	*p* Value	Adj. *R^2^* (%)	β (95%CI)	*p* Value	Adj. *R^2^* (%)	β (95%CI)	*p* Value	Adj. *R^2^* (%)
Total BCT	−0.03 (−0.10, 0.05)	0.475	0	−3.99 (−10.5, 2.48)	0.202	21.6	−0.04 (−0. 08, 0.01	0.107	30.12
Behaviour strategies consistent with control theory	0.46 (−0.48, 1.40)	0.323	0	−20.4 (−230.5, 189.8)	0.835	0	−0.26 (−0.90, 0.39)	0.413	0
Goal setting (behaviour) (1.1)	−0.06 (−0.78, 0.66)	0.867	0	−20.4 (−230.5, 189.8)	0.835	0	−0.26 (−0.90, 0.39)	0.413	0
Problem solving (1.2)	−0.18 (−0.91, 0.54)	0.604	0	−62.2 (−163.9, 39.0)	0.203	24.2	−0.04 (−0.41, 0.33)	0.817	0
Goal setting (outcome) (1.3)	−0.29 (−0.96, 0.38)	0.383	10.6	−24.2 (−124.5, 76.0)	0.605	0.11	−0.11 (−0.48, 0.26)	0.647	0
Action planning (1.4)	−0.09 (−0.78, 0.60)	0.795	0	−18.9 (−132.1, 94.2)	0.720	0	−0.06 (−0.47, 0.35)	0.763	0
**Review behaviour goals (1.5)**	−0.65 (−1.34, 0.08)	0.079	27.5	−90.6 (−164.6, −16.7)	0.021	52.6	−0.15 (−0.52, 0.22)	0.405	0
Discrepancy between current behaviour and goal (1.6)	−0.68 (−1.69, 0.33)	0.179	15.4	−60.4 (−135.2, 14.4)	0.103	43.4	NA	NA	NA
Review outcomes goals (1.7)	−0.38 (−1.43, 0.67)	0.462	5.33	−24.3 (−119.6, 71.1)	0.587	0	NA	NA	NA
**Feedback on behaviour (2.2)**	−0.73 (−1.43, −0.03)	0.042	40.1	−61.7 (−136.6, 13.1)	0.097	43.9	−0.05 (−0.47, 0.37)	0.798	0
Self-monitoring of behaviour (2.3)	−0.54 (−1.19, 0.11)	0.103	20.6	−20.9 (−117.6, 75.6)	0.642	0	0.22 (−0.11, 0.57)	0.174	21.12
Self-monitoring of outcomes of behaviour (2.4)	0.76 (0.16, 1.14)	0.015	29.7	6.3 (−91.4, 103.9)	0.890	0	0.09 (−0.32, 0.51)	0.644	0
Feedback on outcomes of behaviour (2.7)	0.61 (−0.18, 0.14)	0.123	25.6	NA	NA	NA	−0.36 (−0.74, 0.01)	0.053	34.2
Social support (unspecified) (3.1)	−0.32 (−1.02, 0.38)	0.350	8.2	26.2 (−61.7, 114.1)	0.525	0	0.17 (−0.21, 0.56)	0.358	0
Social support (emotional) (3.3	−0.65 (−1.55, 0.26)	0.156	21.1	NA	NA	NA	−0.15 (−0.62, 0.32)	0.503	0
Instruction on how to perform the behaviour (4.1)	0.08 (−0.61, 0.78)	0.813	0	−33.9 (−119.5, 51.8)	0.403	0	0.04 (−0.35, 0.44)	0.811	0
Information about health consequences (5.1)	0.15 (−0.67, 0.98)	0.709	0	−21.9 (−114.2, 70.5)	0.623	0	−0.31(−0.70, 0.09)	0.117	20.4
Information about social and environmental consequences (5.3)	0.71 (0.05, 1.37)	0.037	23.9	NA	NA	NA	−0.04 (−0.56, 0.44)	0.865	0
Demonstration of the behaviour (6.1)	0.00 (−0.87, 0.87)	0.998	0	−59.7 (−140.3, 20.9)	0.131	43.3	−0.07 (−0.52, 0.38)	0.750	0
Behaviour practice/rehearsal (8.1)	0.13 (−0.74, 1.01)	0.758	0	−0.81 (−102.2, 100.6)	0.986	0	−0.01 (−0.69, 0.68)	0.982	0
Behaviour substitution (8.2)	−0.54 (−1.33, 0.25)	0.171	22.0	NA	NA	NA	NA	NA	NA
**Graded tasks (8.7)**	−0.82 (−1.46, −0.17)	0.015	50.3	NA	NA	NA	0.45 (−0.04, 0.94)	0.070	32.72
Credible source (9.1)	−0.24 (−0.96, 0.49)	0.510	0	52.7 (−50.5, 155.9)	0.285	14.2	0.09 (−0.28, 0.46)	0.611	0
Reward (outcome) (10.10)	NA	NA	NA	−60.3 (−135.2, 14.6)	0.104	43.4	NA	NA	NA
Reduce negative emotions (11.2)	0.35 (−0.53, 1.23)	0.421	0	NA	NA	NA	0.03 (−0.47, 0.53)	0.894	0
Adding objects to the environment (12.5)	−0.46 (−1.15, 0.23)	0.185	15.4	NA	NA	NA	0.05 (−0.38, 0.48)	0.754	0

β = beta coefficient; CI = confidence interval; *n* = number of studies; NA = not applicable because a BCT is not present in at least three studies; Adj. *R^2^* = adjusted *R^2^* which measures percentage of variation. BCTs in bold text denote significant association.

**Table 2:** Percentage of behaviour change techniques used in effective and non-effective interventions for weight, energy intake and physical activity.

Behaviour Change Strategies	Weight (*n* = 29)		Energy Intake (*n* = 13)				Physical Activity (*n* = 17)		
	*Effective*	*Non-* *Effective*	*Percentage* *of Effectiveness*	*Effective*	*Non-* *Effective*	*Percentage* *of Effectiveness*	*Effective*	*Non-* *Effective*	*Percentage* *of Effectiveness*
**Goal setting (behaviour) (1.1)**	11	8	**57.9**	4	6	40.0	3	12	20.0
**Problem solving (1.2)**	6	4	**60.0**	4	3	**57.1**	2	6	25.0
**Goal setting (outcome) (1.3)**	8	3	**72.7**	3	2	60.0	1	5	16.7
**Action planning (1.4**	8	4	**66.7**	3	4	42.9	2	9	18.2
**Review behaviour goals (1.5)**	4	3	**57.1**	4	2	**66.7**	2	4	33.3
**Discrepancy between current behaviour and goal (1.6)**	3	0	**100**	2	1	**66.7**	NA	NA	NA
**Review outcomes goals (1.7)**	NA	NA	NA	2	1	**66.7**	NA	NA	NA
**Feedback on behaviour (2.2)**	4	1	**80.0**	3	1	**75.0**	1	3	25.0
**Self-monitoring of behaviour (2.3)**	12	5	**70.6**	2	3	40.0	2	7	22.2
**Self-monitoring of outcomes of behaviour (2.4)**	7	5	**58.3**	1	2	33.3	1	3	25.0
Feedback on outcomes of behaviour (2.7)	1	2	33.3	NA	NA	NA	0	3	0
**Social support (unspecified) (3.1)**	11	2	**84.6**	1	3	25.0	2	4	33.3
**Social support (emotional) (3.3)**	3	1	**75.0**	NA	NA	NA	1	3	25.0
**Instruction on how to perform the behaviour (4.1)**	8	6	**57.1**	4	6	40.0	3	9	25.0
**Information about health consequences (5.1)**	6	1	**85.7**	2	1	**66.7**	0	3	0
Information about social and environmental consequences (5.3)	2	3	40.0	NA	NA	NA	0	3	0
**Demonstration of the behaviour (6.1)**	3	1	**75.0**	2	2	50.0	1	2	33.3
**Behaviour practice/rehearsal (8.1)**	2	2	50.0	2	1	**66.7**	NA	NA	NA
**Behaviour substitution (8.2)**	4	1	**80.0**	NA	NA	NA	NA	NA	NA
**Graded tasks (8.7)**	5	0	**100**	NA	NA	NA	1	2	33.3
**Credible source (9.1)**	8	4	**66.7**	0	5	0	3	5	37.5
**Reward (outcome) (10.10)**	NA	NA	NA	3	0	**100**	NA	NA	NA
Reduce negative emotions (11.2)	2	5	28.6	NA	NA	NA	0	4	0
**Adding objects to the environment (12.5)**	4	2	**66.7**	NA	NA	NA	0	4	0

BCT is considered effective if identified in a significant effect size of outcomes (weight, energy intake and physical activity); NA = not applicable because a BCT is not present in at least three studies. BCTs in bold text denote that had a percentage effectiveness ratio >50%.

## Supplementary Materials

The following are available online at https://www.mdpi.com/article/10.3390/nu14010209/s1, Figure S1: Preferred Reporting Items for Systematic Reviews and Meta-analysis flow diagram of included studies., Table S1: Intervention and comparator characteristics of included studies, Figure S2: Forest plot for physical activity.
