# Supplementary material for: Behaviour Change Techniques in Weight Gain Prevention Interventions in Adults of Reproductive Age: Meta-Analysis and Meta-Regression
Source: Nutrients. 2022 Jan 3;14(1):209. doi: 10.3390/nu14010209 (PMC8747167; doi:10.3390/nu14010209)
Supplement: Supplementary file 1 [file nutrients-14-00209-s001.zip › nutrients-1510557-supplementary.pdf]

Supplementary materials

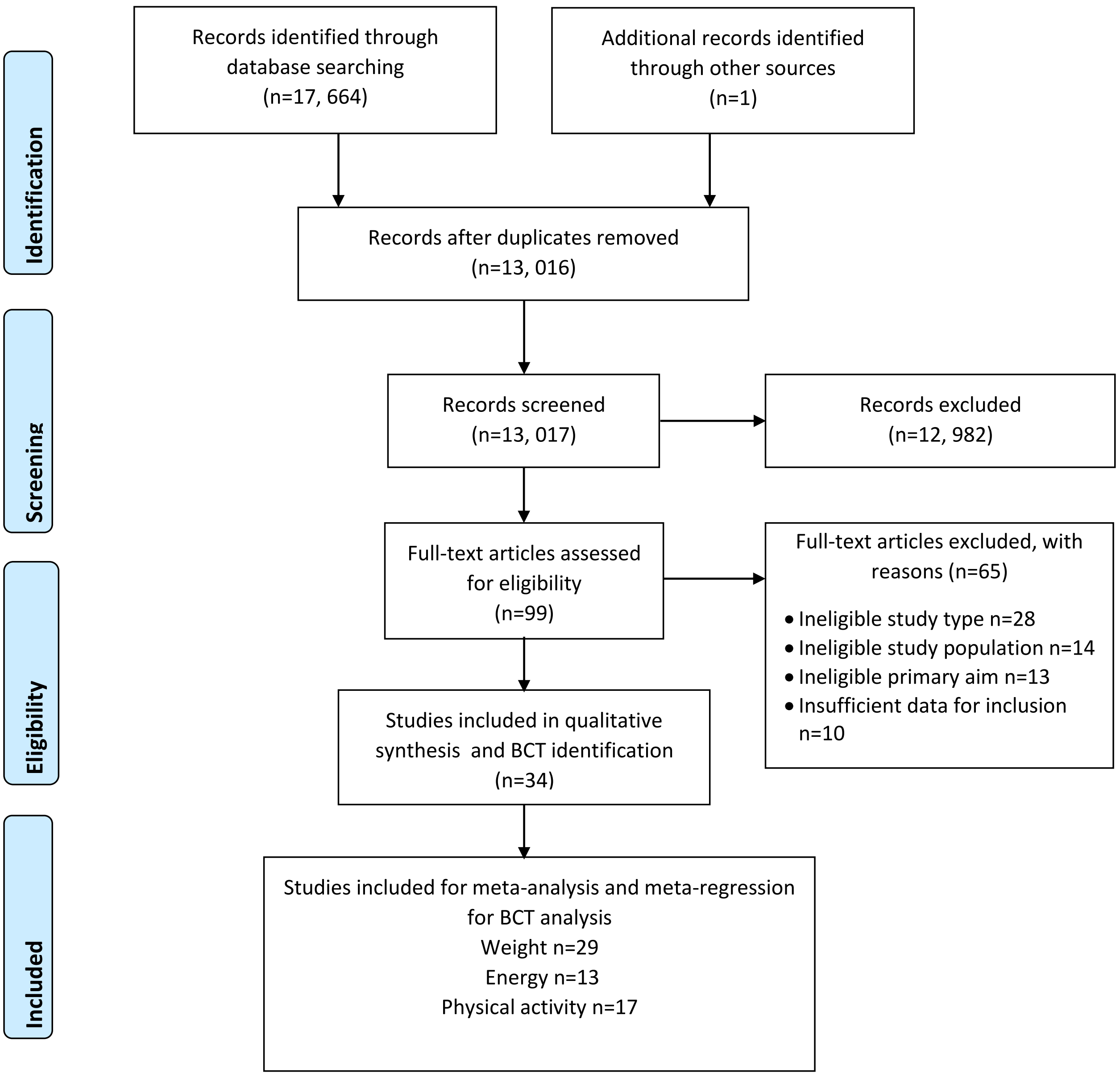

Figure S1. Preferred Reporting Items for Systematic Reviews and Meta-analysis flow diagram of included studies for meta-analysis and meta-regression.

BCT: Behaviour change techniques
Adapted from Martin et al., 2021 as a secondary analysis of a previous systematic review

Table S1. Intervention and comparator characteristics of included studies.

First author, year, country, N = number randomized	Intervention	Comparator
Bennett et al. 2013; Foley et al. 2012, USA N=194	Behavioural: Weekly interactive voice response calls, behavioural goals, annual gym membership. Intensity: monthly calls Facilitator: trained registered dietitian	Newsletters: health topics (weight, nutrition, and PA excluded) sent bi-yearly
Bertz *et al.* 2015, USA N=167	Calorie titration method. Daily e-mailed graph of weight to avoid weight gain above target weight.	Emailed contact for weight 6 monthly, no feedback provided
Chang *et al.* 2017; Chang et al.2014 USA N=612	Behavioural: Theory-based culturally sensitive interactive DVDs and peer support group teleconferences Intensity: Week 1-4 weekly, week 5-16, every other week. Teleconference (5x30 min sessions) Facilitator: Counselling (masters), with motivational interviewing	Handouts: stress management, healthy eating, PA. DVD about food and home safety
Chang *et al.* 2010 USA, N=129	Behavioural: Theory-based culturally sensitive interactive DVDs and peer support group teleconferences Intensity: Week 1-4 weekly, week 5-16, every other week. Teleconference (5x30 min sessions) Facilitator: Counselling (masters), with motivational interviewing	Nutrition education sessions: 6 months
Cheung *et al.* 2017, The Netherlands, N=2423	Behavioural: Web-based tailored behavioural change program: video or text format. Intensity- 6 weekly sessions X 15 mins	No intervention
Donnelly *et al.* 2003, USA N= 131	PA: Tailored supervised exercise program based on baseline maximal treadmill test updated 4 monthly. Intensity: Baseline (20 mins), 6 months (45 mins, 60% heart rate reserve at baseline to 75% at 6 months Facilitator: research assistant	Usual PA and diet
Eiben *et al.* 2006, Sweden N=40	Behavioural: Tailored support package (PA, diet, weight), baseline counselling session, regular phone/email contact, occasional group sessions, special interest lectures, dietitian visits. Intensity: mailed package; fortnightly, follow up calls; monthly Facilitator: Dietitian	Delayed treatment control group
Forster *et al.* 1988, USA, N=219	Diet and PA: Newsletter containing weight control topics. Financial incentive for weight maintenance, 4 session weight management course (optional for weight gainers) Intensity: monthly	No intervention
Gow *et al.* 2010, USA, N=170	Behavioural: Three groups: Internet: online group sessions on diet, PA, behavioural change. Feedback: self-reported weight, e-mailed graph of change in weight with equivalent caloric change. Combined: received both feedback and internet Intensity: 6 weekly X 45 mins Facilitator: trained clinician	No intervention

Hivert *et al.* 2007<br>Canada, N=115	Behavioural: Group sessions: complications of weight gain, PA, diet<br>Intensity: Fortnightly for 2 months, then monthly ~45 min sessions<br>Facilitators: Endocrinology resident, PA graduate	No intervention
Jeffery *et al.* 1999<br>USA, N=1226	Diet & PA/mixed: Healthy lifestyle/ behavioural change newsletter, return quiz, low cost activities<br>Two formats: Education only and Education plus incentive<br>Education plus incentive: financial incentive on returning quiz<br>Intensity: Monthly newsletter, 6 monthly activities	No intervention
Katterman *et a*l 2014,<br>USA, N=58	Behavioural: Face-to face group behavioural change sessions<br>Intensity: 8 sessions X 75 mins, sessions 4-5 weekly, then monthly<br>Facilitator: Graduate students with behavioural weight loss experience	No intervention
Klem *et al.* 2000<br>USA, N=102	Diet & PA/mixed: Healthy lifestyle course: behavioural skills with individual weight, calorie and fat goals<br>Delivery format: (Group or Correspondence)<br>Intensity: 10 weekly sessions	Lifestyle brochure
LaRose *et al* 2019<br>USA, N= 609	Diet & PA/mixed: Prescribed diet and exercise plans.<br>Small changes (SC): deficit of 100 kcal/day, steps increased to 2000/day<br>Large changes (LC): deficit of 500-1000 kcal/d and increasing moderate to vigorous PA to ≥250 mins/week, All participants daily self-weighing with feedback. Intensity: 10 × group sessions, weeks 1-8 weekly, weeks 9-16 monthly. 2× 4-weekly online refresher campaigns, monthly remote weight reporting, quarterly newsletter<br>Facilitator: health science (masters) and behavioural weight management training	1 group session: weight gain, an overview of interventions. Participants chose an intervention. Quarterly newsletter, monthly personalised feedback on weight
Leermakers *et al.* *1998,* USA, N=67	Diet & PA/mixed: Prescriptive diet and exercise program with behavioural change strategies.<br>Clinic: supervised face to face group sessions<br>Home-based: correspondence with health coaching calls<br>Intensity: Clinic- 30 minute × 8 weekly sessions, then biweekly thereafter<br>Home-1 session, weekly newsletters for 8 weeks, weekly/biweekly telephone calls<br>Facilitator: Exercise physiologist, dietitian, behavioural therapist	No intervention
Lemon *et al* 2014<br>USA, N=867	Diet & PA/mixed: A multilevel program targeting the nutrition and physical activity environment, policies, and individual knowledge, attitudes and skills<br>Intensity: quarterly meetings<br>Facilitator: Coaches, school employees, employee advisory group	Employee resource book: healthy eating, PA and weight management, website access, weekly newsletter
Levine *et al.* 2007<br>USA, N= 284	Diet & PA/mixed: Prescriptive diet and exercise program with behavioural change strategies. Clinic: Face to face group meetings<br>Correspondence: mailed format with returned homework assignments<br>Intensity: 15 meetings, biweekly for 2 months and bimonthly thereafter<br>Facilitator: trained nutritionists, behavioural interventionists	Booklet: benefits of weight maintenance, low-fat eating, regular PA

Lombard *et al.* 2010, Australia N=250	Behavioural: Face to face group sessions: simple health messages, behaviour change strategies. Pedometers: 8000-10,000 steps/d<br>Intensity: 4 × 1-hour group sessions. Monthly SMS. Motivational flyer x1<br>Facilitator: Dietitian	One information session: population diet and PA. Pedometer provided: no step goal
Lombard *et al.* 2016, Australia N= 649	Behavioural: Face to face group session: simple health messages, behaviour change manual<br>Intensity: 1x 60 min group session, 1 x20 min phone call. Monthly SMS.<br>Facilitator: staff trained in motivational interviewing	Group session x1: women's health
Lombard *et al.* 2009, Australia N= 250	Behavioural: Face to face group sessions: simple health messages, behaviour change strategies. Pedometer:8000-10,000 steps/d<br>Intensity: 4 × 1-hour group sessions, monthly SMS, calls or email<br>Facilitator: Dietitian	Group session x1: population diet and PA. Pedometer provided: no goal
Lytle *et al.* 2017 USA, N=441	Behavioural: College course: diet, PA, stress management, sleep. Optional online, face-to-face or hybrid version. Access to social network website. Points for participation were provided and redeemed for wellness products	Health assessments and measurement, quarterly health information
Mason *et al.* 2018 UK, N=272	Behavioural: Self-monitor weight, reflect on weight trajectory; 10 tips for weight management, pictorial information of PA calorie equivalent of festive foods/beverages<br>Intensity: record weight min 2/weekly, ideally daily	Brief leaflet: healthy lifestyle
Matvienko *et al.* 2001, USA, N=40	Diet: College course: scientific principles related to weight gain prevention<br>Intensity: twice weekly comprising lectures and laboratory exercises ~50 mins each<br>Facilitator: lecturer	No intervention
Medina *et al* 2016 USA, N=119	Diet & PA/mixed: Group sessions: prescriptive diet and exercise plans, behavioural change strategies<br>Intensity: 10 weekly group sessions ~90 mins each<br>Facilitator: clinical psychology student or trained undergraduate research assistants	Delayed treatment control group
Metzgar *et al.* 2016, USA, N=87	Behavioural: Face to face group sessions: nutrition education, lifestyle behaviour<br>Intensity: 24 × 1 hr sessions. Months 1-4 weekly sessions, months 5-12 monthly sessions<br>Facilitator: Registered dietitian and counsellor	No intervention
Middleton *et al* 2014, USA, N=95	Diet & PA/mixed: Face to face group sessions: prescribed diet and exercise plans, behavioural change strategies<br>Intensity: 5X weekly sessions<br>Facilitator: Clinical psychology student	Waist list control
Nikolaou *et al* 2015, Scotland UK, N= 20,975	Diet & exercise/mixed: Online healthy lifestyle courses: two treatments; "Not the ice-cream van (NTICV)": unwanted weight gain and obesity,	No intervention

	"Goddess Demetra (GD)": social and political movements associated with diet/lifestyle<br>Intensity- weekly	
Partridge *et al* 2016, Australia, N=250	Behavioural: Health coaching calls, SMS, emails, smartphone application and website.<br>Intensity: 5 × health coaching calls (10-15 mins), 8 × SMS (2 per week), weekly emails, booklet containing diet, PA information<br>Facilitator: dietitian	Brochure: diet and PA; introductory call (no coaching), 4 × SMS
Rodearmel *et al.* 2006, USA, N= 159	Diet & PA/mixed: Prescriptive diet and exercise program: walking and cereal consumption targets. Step counters, calculators, cereal provided	Usual eating and step patterns. Step counters, calculators provided
Verweij *et al.* 2012, The Netherlands N=28 OPs N= 523	Behavioural: Guideline based care provided by OP's to employers and employees. Advice at the environmental and individual level<br>Intensity: 5 × counselling sessions (~20-30 mins)<br>Facilitators: OPs with behaviour change counselling	OPs provided usual care.
Walthouwer *et al* 2015, The Netherlands, N=1419	Behavioural: Web-based individually tailored behavioural change program: video or text format<br>Intensity- 6 weekly sessions, 15 mins each	No intervention
Wing *et al* 2016 USA, N=609	Diet & PA/mixed: Prescribed diet and exercise plans.<br>Small changes (SC): deficit of 100 kcal/day, steps increased to 2000/day<br>Large changes (LC): deficit of 500-1000 kcal/d and increasing moderate to vigorous PA to ≥250 mins/week. All participants daily self-weighing with feedback. Intensity: 10 × group sessions, weeks 1-8 weekly, weeks 9-16 monthly. 2× 4-weekly online refresher campaigns, monthly remote weight reporting, quarterly newsletter<br>Facilitator: health science (masters) and behavioural weight management training	1 group session: weight gain, an overview of interventions.<br>Participants chose an intervention. Quarterly newsletter, monthly personalised feedback on weight
Williams *et al* 2014, Australia N=54	Diet & PA/mixed: late perimenopause.<br>Face to face counselling sessions, individualised weight management booklet. Intensity: 5 sessions x 1 hour.<br>Facilitators: Dietitian, exercise physiologist with motivational interviewing	Weight management booklet, baseline anthropometric, biochemistry, dietary and PA results to assist self-monitoring of weight, and tailored health goals
Williams *et al* 2019, Australia N=54	Diet & PA/mixed: late perimenopause.<br>Face to face counselling sessions, individualised weight management booklet. Intensity: 5 sessions x 1 hour.<br>Facilitators: Dietitian, exercise physiologist with motivational interviewing	Weight management booklet, baseline anthropometric, biochemistry, dietary and PA results to assist self-monitoring of weight, and tailored health goals

Age in years and BMI of participants are reported as mean ± SD at baseline, unless otherwise stated. C: comparator/ control, F: female, M: male, I: intervention, N: number of participants randomized, SD: standard deviation, SE: standard error, PA: physical activity, ow: overweight, OPs: occupational physician.

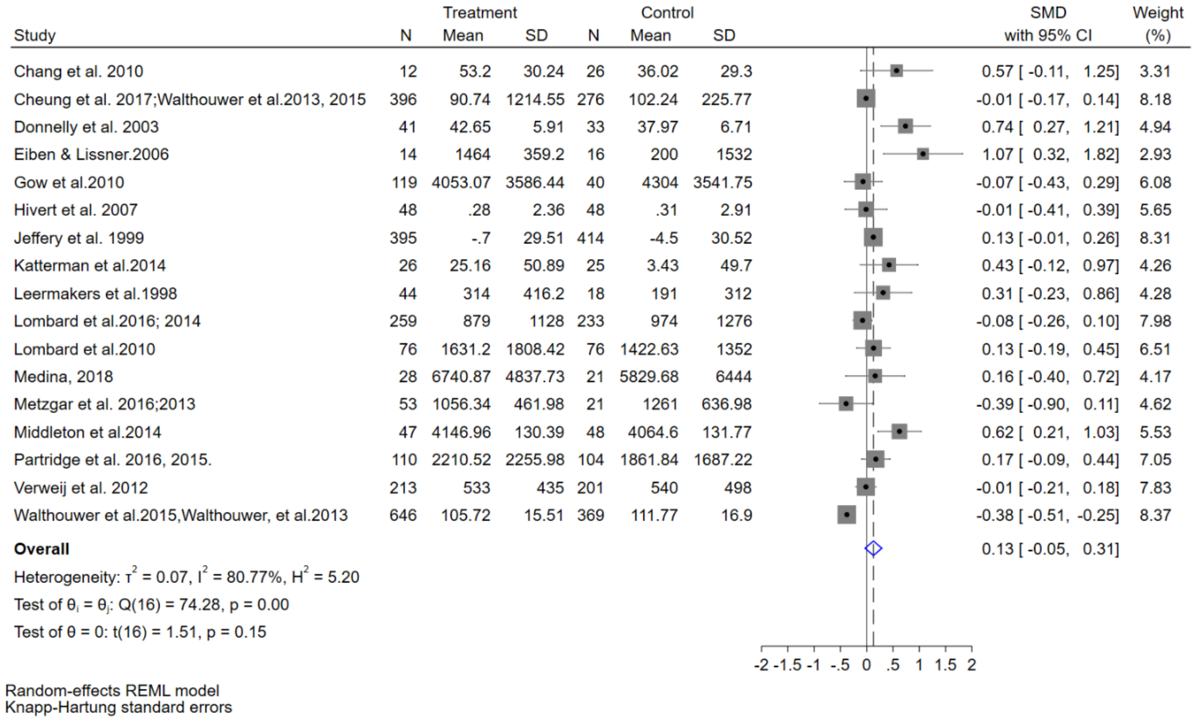

Figure S2: Forest plots for physical activity
